# Recovery of Model Pharmaceutical Compounds from Water and Organic Solutions with Alginate-Based Composite Membranes

**DOI:** 10.3390/membranes12020235

**Published:** 2022-02-18

**Authors:** Tatyana Anokhina, Evgenia Dmitrieva, Alexey Volkov

**Affiliations:** A.V. Topchiev Institute of Petrochemical Synthesis RAS, 29 Leninsky Prospekt, 119991 Moscow, Russia; dmitrievaes@ips.ac.ru (E.D.); avolkov@ips.ac.ru (A.V.)

**Keywords:** sodium alginate, organic solvent nanofiltration, “green” membranes, antibiotics, metal cations

## Abstract

In this work, we combined the non-solvent induced phase separation (NIPS) and further cross-linking by cations towards the preparation of nanofiltration membranes based on sodium alginate, a biodegradable, natural polymer. Acetone, ethanol, toluene, and hexane were used as non-solvents, and cations of calcium, silver, and aluminum—for polymer cross-linking, respectively. Results showed the precipitation strength of non-solvent played a noticeable role in the membrane’s performance; for instance, the toluene permeability changed by four orders of magnitude with the decrease of precipitation strength of the non-solvent: acetone (*P*_toluene_ = 0.1 kg∙m^−2^∙h^−1^∙bar^−1^) < ethanol (3 kg∙m^−2^∙h^−1^∙bar^−1^) < hexane (41 kg∙m^−2^∙h^−1^∙bar^−1^) < toluene (415 kg∙m^−2^∙h^−1^∙bar^−1^). It was shown that simultaneous precipitation and crosslinking in aqueous solutions AlCl_3_ or AgNO_3_ must be used in the preparation of alginate membranes for the highly selective recovery of pharmaceutical compounds from organic media. These membranes show rejection *R* = 90–93% of substances with *MW* = 626 g/mol and ethanol permeability *P*_EtOH_ = 1.5–2.5 kg∙m^−2^∙h^−1^∙bar^−1^. For the highly selective recovery of pharmaceutical compounds from water, the method of obtaining membranes must be changed. Precipitation in toluene and then crosslinking in aqueous solutions of AlCl_3_ or AgNO_3_ must be used sequentially instead of simultaneous precipitation and crosslinking in aqueous solutions of the same inorganic salts. The permeability of such membranes varied from 0.44 to 7.8 kg∙m^−2^∙h^−1^∙bar^−1^ depending on the crosslinking cation in the alginate. The rejection of model substances with *MW* 350 and 626 g/mol were on the level of 99%. Alginate membranes can be used to solve separation problems in the pharmaceutical field, for example, to isolate antibiotics from their extractants and remove the same antibiotics from aqueous pharmaceutical waste to prevent their accumulation in the environment and the emergence of resistant genes and bacteria.

## 1. Introduction

Sodium alginate ((C_6_H_7_O_6_Na)_n_) is a salt form of alginic acid (see Figure S1), a natural polymer extracted from brown algae and also produced by some microorganisms [1]. The molecular weight of sodium alginate ranges from 50,000 to 200,000 and depends on the degree of polymerization, which can reach 750 [2]. The polymer is widespread and is an inexpensive, biodegradable, non-toxic, and water-soluble organic material [3]. Sodium alginate (NaAlg) is traditionally used as a thickener and gelling agent in the food and cosmetic industries [4,5] and in pharmacology [6,7,8,9].

In recent years, sodium alginate has been actively studied as a membrane material for different applications due to its stability in organic solvents and ability to form water-insoluble complexes by introduction of multivalent cations such as aluminum or calcium (see Figure S2) or monovalent cation reduced to the nanoparticles (e.g., silver) [10,11,12,13,14,15,16]. As a result, the membranes based on cross-linked sodium alginate were successfully utilized for separation of water and organic media including pervaporative dehydration of various solvents (alcohols, acetic acid, dioxane, tetrahydrofuran, etc.) [10,11,17,18,19,20,21,22,23,24], as the electrolyte material in the methanol fuel cells [25,26,27], aqueous nanofiltration [28,29,30], and very recently for organic solvent nanofiltration [10,31]. The advantage of natural polymers over the synthetic polymers is their biodegradability which simplifies their utilization from an environmental point of view. Furthermore, unlike the natural polymer cellulose, the processing of sodium alginate into a non-soluble membrane does not usually require the use of organic solvents.

During the synthesis, it is required to isolate the active pharmaceutical ingredients (API) from the reaction mixture. The molecular weight of typical API is withing the range of 170–840 g/mol. Aliphatic alcohols (methanol, ethanol, etc.) and various non-polar aprotic solvents (tetrahydrofuran, methyl ethyl ketone, etc.) can be used as components of the reaction mixture and extractants [32,33,34,35,36]. In recent years, the issue of the accumulation of antibiotics in the environment has been of concern to the World Health Organization (WHO) and the expert scientific community, as it is one of the reasons for the emergence of antibiotic-resistant bacteria resistant and resistant genes, which are increasingly threatening to human health and life [37,38]. Antibiotics enter the environment with wastewater from pharmaceutical and food industries, fish farming and animal husbandry, and household and medical wastewater. However, up to 70% of antibacterial drugs are not absorbed by the human body and are unchanged. In 2020–2021, the coronavirus infection (COVID19) pandemic exacerbated the problem with the accumulation of antibiotics in nature since the treatment protocol for patients included macrolide antibiotics and β-lactam antibiotics to prevent secondary bacterial pneumonia. Thus, today antibiotics are considered a new class of pollutants due to their ubiquitous presence in high concentrations in surface and ground waters, soils, and biota in much of the world [39,40,41].

It was demonstrated [11,31] that the transport and separation characteristics of such membranes can be significantly varied by the changing of the cation nature and cross-linking conditions. However, further tunning of the membrane properties can be achieved by preliminary precipitation of sodium alginate by using organic solvent followed by the cross-linking with different metals. Thus, the goal of this work was to develop alginate-based membranes for the recovery of bulk organic compounds with the molecule size typical for API from its aqueous and organic media. The combined approach for fabrication of alginate-based fibers by using non-solvent-induced phase separation (NIPS) followed by cross-linking was recently reported [13], and it was used in this study for the fabrication of alginate-based membranes for the first time.

## 2. Materials and Methods

### 2.1. Materials

The selective layer of composite membranes was made from natural polymer sodium alginate (Rhône-Poulenc, Dijon, France). Distilled water was used to dissolve sodium alginate. A polyester nonwoven fabric from Crane Technical Materials (Channahon, USA) was used as a support. The density of nonwoven fabric was 87 g/m^2^, thickness 90–99μm, air permeability 16.0–29.6 sm^3^/sm^2^⋅s⋅kPa. Ethanol (ECOS-1, Moscow, Russia), Toluene (ECOS-1, Russia), acetone (HIMMED, Moscow, Russia), and hexane (HIMMED, Russia) were used to precipitate the polymer. Inorganic salts were used as precipitating/crosslinking agents: CaCl_2_∙2H_2_O (HIMMED, Russia), AlCl_3_∙6H_2_O (HIMMED, Russia), and AgNO_3_ (SibProject—DragMet, Moscow, Russia).

Acetone, hexane, toluene, ethanol, and distilled water were used to study the transport properties of the composite membranes. The dyes Remazol Brilliant Blue R with MW 626 and Orange II with MW 350 (see Figure S3) were selected to model the pharmaceutical compounds such as Lomefloxacin (MW 351), Enrofloxacin (MW 359), Globomycin (MW 655), Ceftriaxone (MW 661), or Azithromycin (MW 749).

### 2.2. Characterization of Sodium Alginate

Infrared spectroscopy (IR) of sodium alginate powder were recorded in the attenuated total reflection (ATR) mode on a HYPERION-2000 IR microscope coupled to a Bruker IFS-66v/s Fourier spectrometer (scan 50, Ge crystal, resolution 2 cm^−1^, range 600–4000 cm^−1^).

The ratio of β-D-mannuronic (M) and α-L-guluronic (G) acids in sodium alginate was determined by sequential precipitation of a hydrolyzed solution of sodium alginate [42]. It consists of the mild hydrolysis of alginate in a solution of oxalic acid under microwave radiation with sequential precipitation of α-L-guluronic(G) and β-D-mannuronic(M) acids at pH 2.85 and 1.00, respectively.

The molecular weight distribution (MWD) was determined using gel permeation chromatography (GPC) on Agilent 1260 MDS with refractometric and viscometric detection using two series-connected Polargel M 30 × 7.5 columns at an eluent H_2_O flow of 1 mL/min. The volume of the injected sample was 100 µL. Based on the signal of the refractometric and viscometric detectors, the MWD of the samples was calculated using the universal calibration curve according to the Kuhn–Mark–Houwink equation. Standard PEG samples with a known molecular weight (EasiVial PEG) were used to construct the calibration curve.

The intrinsic viscosity of sodium alginate was determined using an Ubellodeviscometer in a tetraborate buffer with the addition of 0.2 NaCl.

### 2.3. Preparation of Composite Membranes with Sodium Alginate

The composite membranes were fabricated by the deposition of top-layer from 10 wt.% solution of sodium alginate in water on the non-woven porous support made of PET by using the doctor blade with a gap thickness of 200 μm. The membrane was placed in the precipitation bath (acetone, hexane, toluene, or ethanol) for 10 min, and then, the cross-linking of sodium alginate was carried out by followed immersion in the excess of 0.35 mol-eq/L solutions of corresponded inorganic salts (AgNO_3_, AlCl_3_, or CaCl_2_). For comparison, a number of membranes were fabricated without precipitation in the organic solvent by direct immersion in the inorganic salt solution.

### 2.4. Study of Precipitation of Sodium Alginate by Organic Solvents

The viscosity of the composition was determined on a Brookfield DV2T viscometer with an RV-07 spindle (7) at its rotation speed of 12 rpm. Composite membranes were prepared by the method of phase inversion—NIPS (non-solvent induced phase inversion) by contacting the precipitant with a polymer solution. The change of viscosity described the changes of the polymer while it contacted with a non-solvent. The viscosity of a 1 wt.% aqueous sodium alginate composition was determined on a Brookfield DV2T viscometer with a ULA (0) spindle at its rotation speed of 5 rpm. Then, the non-solvents were piecemeal added to the aqueous solution of sodium alginate. The moment of reaching the maximum viscosity was recognized as the moment preceding the complete precipitation of the polymer.

### 2.5. Filtration Experiments

The composite membranes based on sodium alginate were tested in nanofiltration of aqueous and organic solutions in dead-end cells at 10 bar. The mixture in the cells was constantly stirred with magnetic stirrers. The permeate flow was determined by the gravimetric method.

A liquid receiver was installed at the outlet of the cell. The mass of permeate passing through the membrane during the experiment was measured on a Sartorius laboratory balance with a measurement error of 0.001 g. Membrane performance was characterized by liquid permeation, P (1):

(1) $$P=\frac{m}{S\;\cdot \;\Delta t\;\cdot \;\Delta p}$$

where m is the weight of permeate (kg) passed through a membrane with an area S (m^2^) over a time Δt (h), Δp is the pressure drop.

The optical density of solutions was measured with PE-5400UF spectrophotometer (PromEcoLab, Russia). First, the concentrations of model compounds (dyes) in the feed and permeate were determined using the calibration curve; after the rejection R (%) was calculated and used to evaluate the separation characteristics of the membrane:

(2) $$R=\left(1-\frac{C_{p}}{C_{0}}\right)\;\cdot \;100\%$$

C_p_ and C_0_ are the dye concentrations in the permeate and feed, respectively.

### 2.6. Sorption Experiments

Sorption measurements were conducted by placing membrane samples in selected solvents for several days after preliminary weighing of the samples. After soaking in solvents, the membranes were taken out, the excess solvent from the surface of the samples was removed with filter paper, and the membranes were weighed daily. The measurements were stopped when the membrane mass did not change for 2 days. The sorption (K_s_) was calculated by the following Equation (3):

(3) $$K_{s}=\frac{\left(m_{1}-m_{o}\right)}{m_{0}}$$

where m_0_ and m_1_ are the masses of the dry and swollen samples, respectively.

### 2.7. X-ray Diffraction (XRD)

X-ray diffraction (XRD) studies of films were performed on a Rigaku diffractometer, Japan. Experimental diffractograms were obtained using an X-ray source with a rotating copper anode Rotaflex RU-200. The operating mode of the source was 50 kV–100 mA. The source was equipped with a horizontal wide-angle Rigaku D/Max-RC goniometer and a secondary graphite monochromator (the wavelength λ of the monochromatic radiation was 1.542 Å). Film samples were fixed on aluminum frames; in this case, scanning was performed in the reflection mode. It should be noted that with the X-ray wavelength used in the reflection mode, the beam scanned the entire depth of the films.

### 2.8. Scanning Electron Microscope (SEM)/Energy-Dispersive Elemental Spectroscopy (EDX)

The morphology of the samples of supports and composite membranes (surface and transverse cleavages) was studied using a scanning electron microscope (SEM) Thermo Fisher Phenom XL G2 Desktop SEM (USA), equipped with a module for energy-dispersive elemental spectroscopy (EDX). Using a magnetron sputter Cressington 108 auto Sputter Coater (UK), a thin layer of gold 5–10 nm was applied to the surface of the samples. The value of the accelerating voltage during the measurement was 15 keV.

## 3. Results and Discussion

### 3.1. Characterization of Sodium Alginate

**Figure 1:**
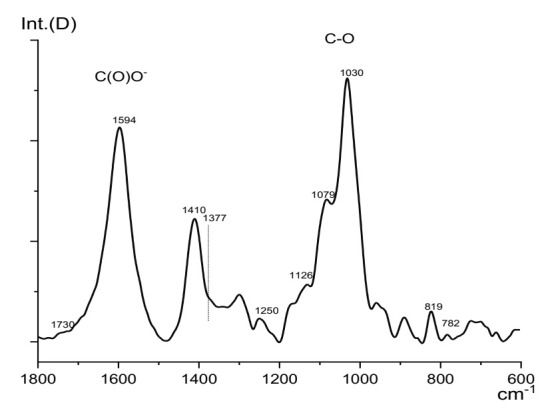
Infrared spectra of sodium alginate: 819 cm^−1^—β-D-mannuronic (M) acid, 782 cm^−1^—α-L-guluronic (G) acid.

The ratio of β-D-mannuronic (M) and α-L-guluronic (G) acids equal to M/G = 5.5 was obtained by the precipitation method described in [42]. As can be seen, the infrared spectra (Figure 1) confirmed the high M/G ratio since the absorption band of the valence vibrations of 819 cm^–1^ (β-D-mannuronic (M) acid) was much more intense than that of 782 cm^–1^ (α-L-guluronic (G) acid). The molecular weight of the polymer and its polydispersity index was determined as *M*_w_ = 1.2 × 10^6^ g/mol and *M*_n_/*M*_w_ = 6.6 by using the gel penetration chromatography. The intrinsic viscosity of the sodium alginate was 3.85 sm^3^∙g^−1^, and the viscosity of 10 wt.% casting solution of sodium alginate in water was 245,000 mPa∙s.

### 3.2. Study of Precipitation of Sodium Alginate by Organic Solvents

**Figure 2:**
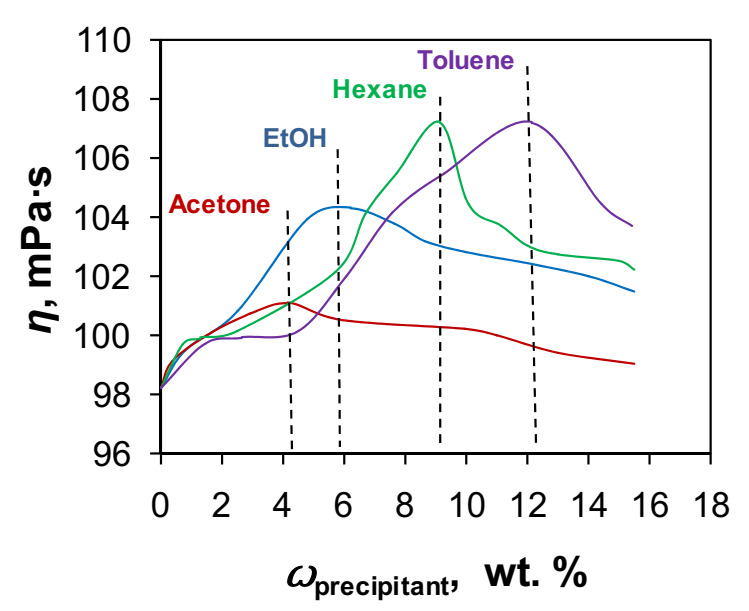
The effect of organic solvent on the viscosity of 1 wt.% aqueous solution of sodium alginate during its precipitation.

Since the sodium alginate was precipitated by forming transparent gel, it was not possible to use tradition technique with “cloud point” to determine the coagulation index. Thus, the precipitation strength of selected organic solvents was studied by measurement of the viscosity of aqueous solution of sodium alginate with stepwise addition of corresponded solvent (acetone, hexane, toluene, and ethanol). Due to high viscosity of casting solution of 10 wt.% (245,000 mPa∙s), the sodium alginate solution of 1 wt.% (98 mPa∙s) was used. Figure 2 illustrates the effect of organic solvent content on the resulted solution viscosity. As can be seen, the viscosity of the solution increased with the addition of organic solvent, reaching its maximum values—101.0 mPa∙s at 4.1 wt.% of acetone, 104.0 mPa∙s at 5.8 wt.% of ethanol, 107.2 mPa∙s at 9.0 wt.% of hexane, and 107.0 mPa∙s at 12.2 wt.% of toluene.

The initial increase of the viscosity can be explained by the increasing of number of interchain contacts due to increasing concentration of “poor” solvent. The highest values of viscosity can be attributed to the maximum concentration of non-solvent before the phase inversion of polymeric solution takes place. In this regard, it can be concluded that the precipitation strength of selected organics solvents is changed in the following order: acetone (4.1 wt.%) > ethanol (5.8 wt.%) > hexane (9.0 wt.%) > toluene (12.2 wt.%). The interactions in the “polymer–solvent–non-solvent” system can also be considered in terms of the solubility parameters of the individual components (Table 1).

It was earlier reported [13,46] that sodium alginate (NaAlg) is soluble in solvents with the Hildebrand parameter (*δ*_t_) > 37 MPa^1/2^; thus, Table 1 shows that only water among selected solvents can be considered as a “good” solvent for this polymer. The Hansen solubility parameters can justify the choice of precipitants. It consists of three components: the dispersion component (*δ*_d_), the polar component (*δ*_p_), and the hydrogen bond component (*δ*_h_) and is calculated by the formula:

(4) $$\delta_{t}=\sqrt{({\left(\delta_{d}\right)}^{2}}+{\left(\delta_{p}\right)}^{2}+{\left(\delta_{h}\right)}^{2})\;$$

Only the complete Hansen solubility parameter *δ*t is known from the literature for NaAlg; to date, the components *δ*d, *δ*p, and *δ*h have not been estimated [23,44,45]. Nevertheless, the general values of the Hansen parameters are sufficient for the selection and evaluation of organic solvents suitable for the precipitation of sodium alginate. For example, Δ*δ*_t_ for NaAlg and acetone (17.8), hexane (22.8), and toluene (14.9) are greater than Δ*δ*_t_ NaAlg for water (10.8); therefore, all the listed organic solvents are precipitants for sodium alginate. For the NaAlg—EtOH pair, Δ*δ*_t_ = 11.3 is comparable to water. However, the hydrogen bond component of ethanol (*δ*_h_ = 19.4 MPa^1/2^) is very low compared to water (*δ*_h_ = 42.3 MPa^1/2^). Since alginate contains many polar hydroxyl and carboxyl groups in which hydrogen is bonded to a strongly electronegative atom and has a partial positive charge, hydrogen bonding is the main mechanism by which sodium alginate dissolves in water. In this regard, ethanol cannot act as a solvent and is a precipitant for alginate [13,42].

### 3.3. Effect of Non-Solvent on Precipitation and Properties of Sodium Alginate

The composite membranes were fabricated by the deposition of sodium alginate layer on the polyester porous substrate. The resulted composite membranes after coagulation bath revealed the pronounced effect of non-solvent nature. In the case of toluene and hexane, the sodium alginate layer was in the form of gel (see Table 2); whereas the sodium alginate precipitated by water or ethanol can be easily handled as polymeric film. Besides, despite the fact that the same width of doctor blade of 200 µm was used for the deposition of 10 wt.% polymeric solution, the thickness of swollen layer of sodium alginate was varied in the wide range, and it was changed in the opposite order of precipitation strength of non-solvent used (see Table 2): acetone (40 µm) > ethanol (55 µm) > hexane (80 µm) > toluene (110 µm).

The obtained membranes were tested for the filtration of ethanol, toluene, hexane, and acetone. As can be seen from Table 2, the solvent permeability was varied by four orders of magnitude (0.1–415 kg∙m^−2^·h^−1^·bar^−1^) with the respect of organic solvent used during the membrane formation and filtration. Such difference was in a good agreement with the solvent quality towards sodium alginate. For instance, the greatest difference in permeabilities was observed for the membranes precipitated by toluene (*P*_toluene_ = 415 kg∙m^−2^·h^−1^·bar^−1^, *P*_acetone_ = 28 kg∙m^−2^·h^−1^·bar^−1^), which can be attributed to the looser packaging of polymer chains resulted in greater thickness of top-layer (110 µm). Besides, the interaction of membrane material with the solvent should be also taken into account. As shown from Table 2, the sorption of ethanol and acetone in dried sodium alginate membrane was not observed (<0.05), whereas the sorption of toluene and hexane was 0.24 and 0.11 g/g, respectively.

**Figure 3:**
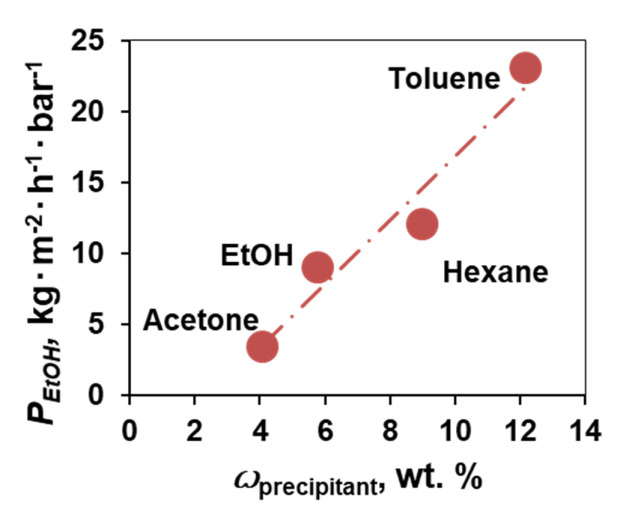
Dependence of ethanol permeability on the amount of organic solvent required for precipitation.

The lowest difference was for the membranes obtained with acetone—0.1–3.7 kg∙m^−2^·h^−1^·bar^−1^ (40 µm). Interestingly, the membrane prehistory played a noticeable role in the filtration experiments. Regardless the solvent used in the filtration experiments, the membranes fabricated with toluene precipitation bath showed the highest permeability values (28–415 kg∙m^−2^·h^−1^·bar^−1^), and the lowest ones were for the membranes precipitated in the acetone bath (0.1–3.7 kg∙m^−2^·h^−1^·bar^−1^). A certain correlation between the permeability of organic solvents through sodium alginate membranes and the amount of non-solvent required for their precipitation can be found. Figure 3 shows the dependence of ethanol permeability through composite membranes obtained by precipitation in toluene, hexane, ethanol, and acetone.

All the membranes based on sodium alginate obtained by using different solvents were characterized by their retention towards two dyes—Remazol Brilliant Blue R (*MW* 625 g/mol) and Orange II (*MW* 350 g/mol). However, the filtration experiments revealed that the retention of these membranes was very low, and did not exceed 10%. Thus, it indicated the necessity of cross-linking this polymer to improve the separation performance.

**Figure 4:**
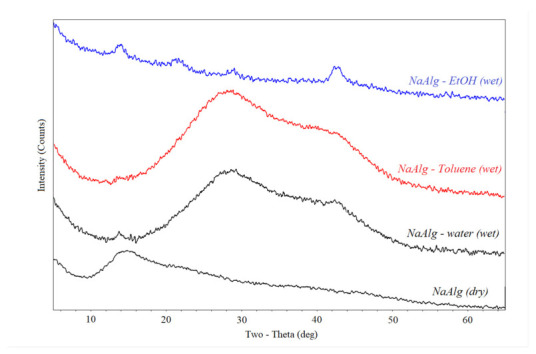
Diffractogram of NaAlg (dry), NaAlg—water (wet), NaAlg—Toluene (wet), NaAlg—EtOH (wet).

To reveal the difference in the structure of the sodium alginate layer in the form of gel (obtained in toluene or hexane) and film (obtained in ethanol and acetone), XRD analysis was used to study the dry NaAlg film, and the films prepared with ethanol, toluene, and water. The XRD diffractogram of dried sodium alginate (Figure 4) consisted of a wide halo (an indicator of amorphous nature) containing a sharp peak at about 2*θ* ~12–14°, indicating a crystalline nature [47,48] with an interchain distance of 6.2–6.9 A [49]. This peak is characteristic of all alginates [50] and shows that polymer chains have a structure centered on a hexagonal lattice. The lattice spacing can be calculated from the observed diffraction signal and is the average distance between the centers of polymer chains [50].

A peak in the region 2θ ~21–23° appeared in the diffractogram for undried sodium alginate precipitated with ethanol. This peak was responsible for structuring the polymer with the formation of a cellular structure [51]. Moreover, in the case of precipitation in toluene, such structuring did not occur. A wide range 2θ ~26–30° and a peak in the region 2θ = 42–4° may indicate the presence of water in sodium alginate precipitated in ethanol and toluene, compared to dried NaAlg. On the other hand, NaAlg precipitated in ethanol had a minimum peak. This was due to the good solubility of water in ethanol and the complete removal of water from the polymer by the organic solvent.

**Figure 5:**
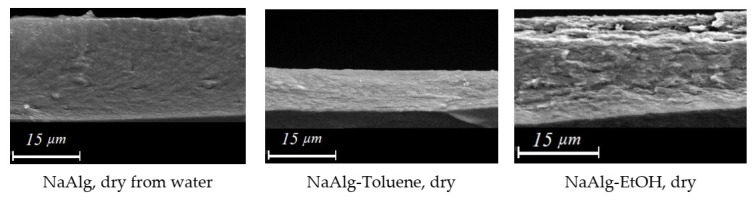
SEM cross-section of selective layer of sodium algimnate precipitated in various solvents after drying and delamination from porous support.

In Figure 5, SEM photographs of a cross-sectional cleavage of a NaAlg layer precipitated in toluene and ethanol and dried from water are presented. The SEM technique does not allow the study of membranes in a wet state. Therefore, samples of NaAlg selective layers of composite membranes were dried after toluene and ethanol. In all cases, a layer of sodium alginate on a polyester substrate was obtained with a doctor blade with the same gap thickness of 200 μm. However, the SEM photographs show the thickness is the same and corresponds to 22 µm for the alginate layer dried from water and precipitated in ethanol. The thickness of the alginate layer is approximately 8–9 µm for dried after toluene. This fact confirms that NaAlg precipitated in toluene and not dried is in a gel form; the evaporation of toluene leads to a strong shrinkage of the polymer layer. Based on the solubility parameters for sodium alginate and the organic solvents (precipitants) used, ethanol is a “soft” precipitant for alginate. The value of its solubility parameter (*δ*_t_ = 26.5 MPa^1/2^) is closest to the value of the solubility parameter of sodium alginate (*δ*_t_ = 37 MPa^1/2^) (Table 2). “Soft” precipitation leads to forming the uniform fine porous layer [52]. The pores collapse when dry. The cross-sectional cleavage of the membrane turns out to be loose, which can be seen in the SEM images (Figure 5). Toluene, on the contrary, is the most “hard” precipitant for alginate (Table 2). “Hard” precipitation leads to forming the denser homogeneous layer (Figure 5).

### 3.4. The Morphology of Selective Layers of Composite Membranes: AgAlg, CaAlg, and AlAlg

It is known from the literature that replacing the Na^+^ cation with other cations leads to an improvement in membrane characteristics [10,11]. In this work, the divalent cation Ca^2+^, the trivalent cation Al^3+^, and the univalent cation Ag^+^ were chosen to improve the transport and separation properties of composite membranes. The composite membranes were obtained by precipitation in organic solvents followed by crosslinking or simultaneous precipitation–crosslinking in 0.35 mol-eq/L of aqueous solutions of inorganic salts. The exchange of cations was observed by qualitative EDX analysis (Table 3). Table 3 shows the Na^+^ cation is completely replaced by another cation, entering into an exchange reaction between sodium alginate and metal salts.

**Figure 6:**
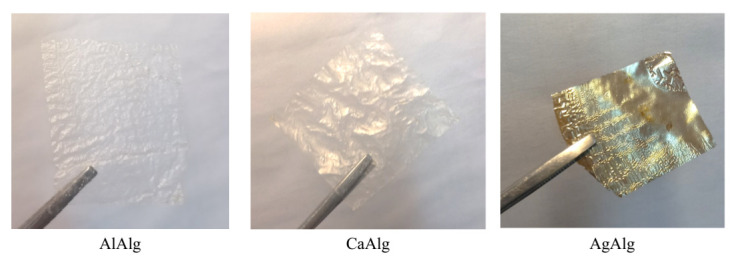
The sodium alginate selective layers cross-linked by different metals after drying and delamination from porous support.

**Figure 7:**
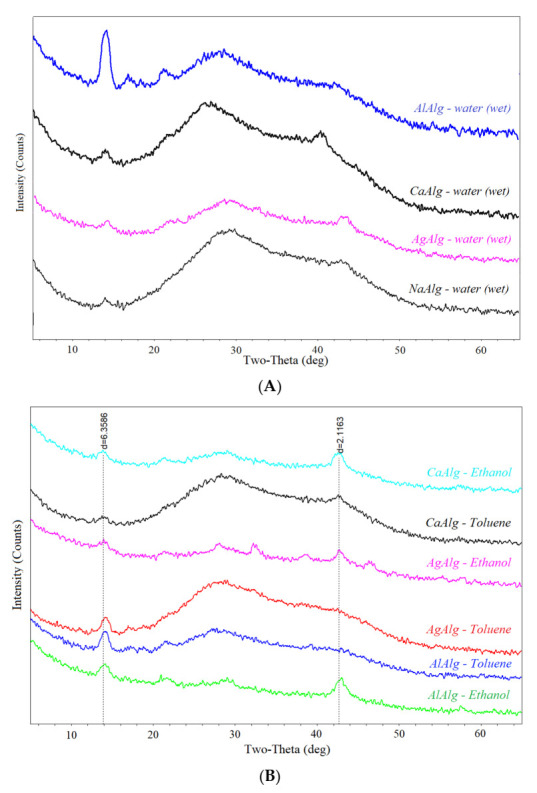
Diffraction pattern of metal alginates: (**A**) simultaneously precipitated and crosslinked with aqueous solutions of metal salts; (**B**) precipitated in organic solvents and crosslinked with aqueous solutions of metal salts.

Dried layers of aluminum and calcium alginate composite membranes are transparent, and silver alginate acquired a silvery-gold color (Figure 6). At the same time, X-ray analysis showed that alginates of various metals have a similar structure when wet (Figure 7).

All investigated alginates have a similar structure, as evidenced by four peaks around 2*θ* ~14°; 22°; 26–30°; 42–44°. The first peak, 2*θ* ~14°^,^ indicates the structuring of the polymer [51]. It is most pronounced in aluminum alginate. It is probably because the aluminum cation is trivalent, and crosslinking with this cation leads to the greatest structuring of alginate chains (Figure 7A). Moreover, the diffraction patterns obtained for selective layers of composite membranes deposited in organic solvents and then crosslinked with metal cations (Figure 7B) are comparable to the diffractograms obtained for the same selective layers of composite membranes but not crosslinked with metal cations (Figure 4).

### 3.5. Nanofiltration Performance of Cross-Linked Membranes

**Figure 8:**
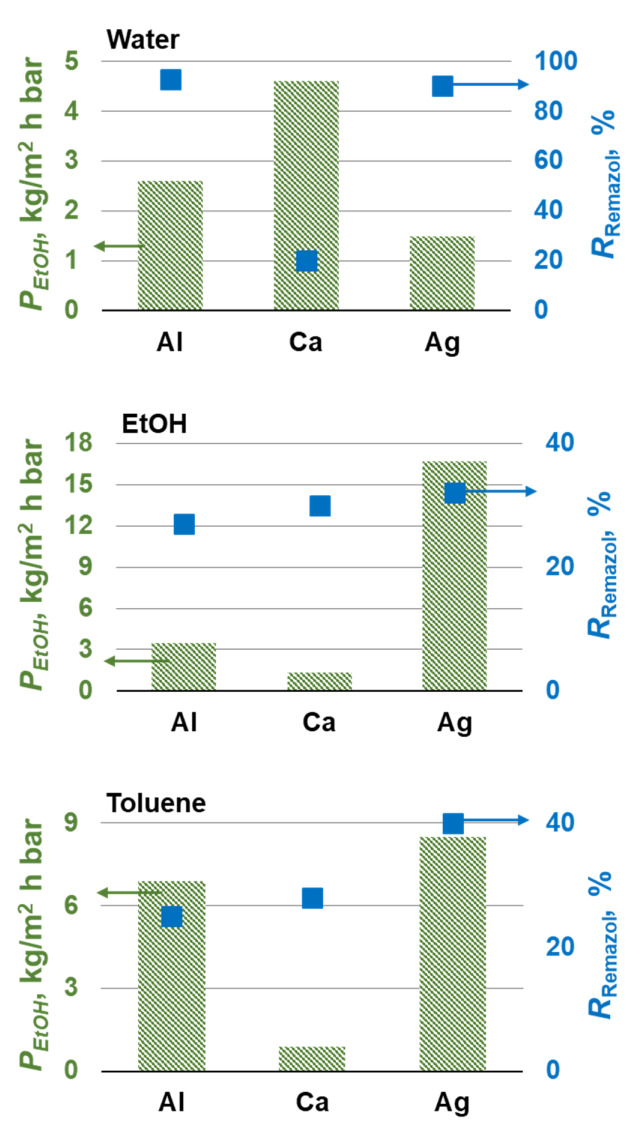
Ethanol transport and organic solutes rejection of the composite membranes based on alginate salts in isolating the model substance from ethanol.

Figure 8 shows that the replacement of the Na^+^ cation with Al^3+^, Ca^2+^, and Ag+ improved the separation properties of composite membranes obtained by precipitation in organic solvents. The best properties were exhibited by composite membranes based on silver alginate. They demonstrated high ethanol permeability of 16.7 and 8.5 kg·m^−2^·h^−1^·bar^−1^ when precipitated in ethanol and toluene, respectively. At the same time, the rejection of the model substance increased from 4 and 6% to 32 and 40%. However, this is not enough for the successful extraction of API from extractants. Therefore, composite membranes were obtained by simultaneous precipitation and crosslinking in aqueous solutions of metal salts. Such composite membranes had a lower ethanol permeability than membranes deposited in organic solvents with subsequent crosslinking. However, composite membranes based on aluminum and silver alginate had high rejection of 93 and 90% of the model dye (*MW* = 626 g/mol), respectively, and *P*_EtOH_ = 1.5–2.5 kg·m^−2^·h^−1^·bar^−1^. These membranes have competitive transport and separating characteristics compared to commercial membranes. Thus, the permeability of commercial membranes MPF-44, MPF-34, M4, Starmem, Solsep to a solution of Methylene Blue (*MW* = 374 g/mol) in ethanol is 0.1, 0.1, 0.5, 4.3, and 7.1 kg·m^−2^·h^−1^·bar^−1^ with a rejection of 95, 94, 97, 43, and 70%, respectively [53].

Thus, it can be concluded that alginate composite membranes obtained by simultaneous precipitation and crosslinking with aqueous solutions of aluminum or silver salts are most promising for the use of organic solvent nanofiltration to isolate antibiotics with a molecular weight of more than 600 g/mol. There is a problem of removing antibiotics from pharmaceutical water effluents to prevent their accumulation in the environment since the emergence of resistant bacteria and genes [37,38]. Composite membranes based on alginate salts were investigated for these purposes, except for NaAlg, since it is soluble in water.

**Figure 9:**
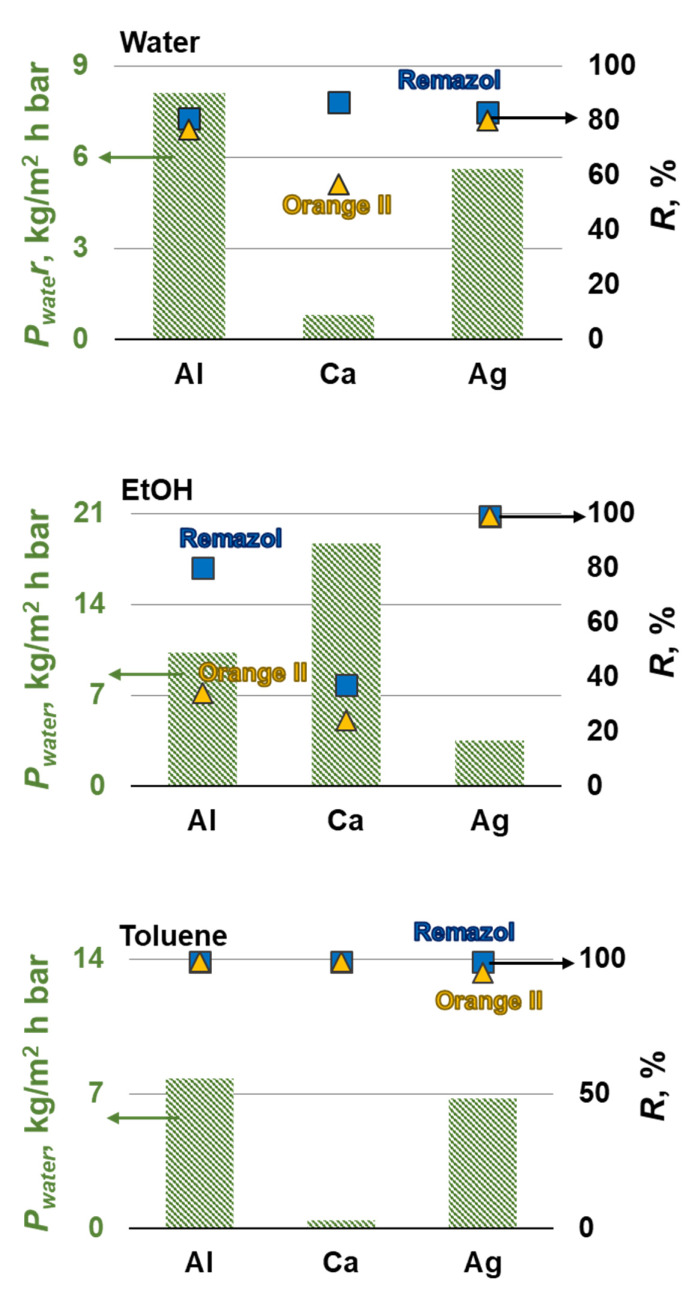
Water transport and organic solutes rejection by composite membranes based on alginate salts in separating model substances from water.

Nanofiltration characteristics are presented in Figure 9. In addition, Remazol Briliant Blue R (*MW* 625) and Orange II (*MW* 350) were also considered as a model substance, as a model of antibiotics (see Figure S5).

Composite membranes deposited in toluene and then crosslinked with metal salts showed the best separation characteristics when the model substances were removed from the water. The Orange II rejection were 95% for silver alginate and 99% for composite membranes based on aluminum and calcium alginates. Remazol rejection was 99% for all composite membranes. Composite membranes based on aluminum alginate had the best water permeability, the value of which was 7.8 kg·m^−2^·h^−1^·bar^−1^. The composite membrane precipitated with ethanol and crosslinked with a silver salt showed good characteristics, *R* for both model substances was 99%, *P*_H2O =_ 3.5 kg·m^−2^·h^−1·^bar^−1^. The membranes obtained in this work have characteristics comparable to alginate membranes, which are known in the literature. Thus, a membrane made from a mixture of polyvinyl alcohol (PVA) and alginate crosslinked with glutaraldehyde has a permeability of 4 kg·m^−2^·h^−1·^bar^−1^ for an aqueous solution of PEG 600 and a rejection *R* = 80% [54]. Thus, the fundamental possibility of using metal alginates to remove antibiotics from pharmaceutical stocks has been shown. Composite membranes deposited in toluene or ethanol and then crosslinked with aluminum or silver salts exhibit the best performance, respectively. In addition, silver has good anti-microbial properties. It makes silver alginate composite membranes promising for removing antibiotics and potential resistant bacteria. Unfortunately, the permeability of obtaining alginate composite membranes is significantly inferior to commercial membranes. Thus, the permeability of the best membranes in this work is 8 kg·m^−2^·h^−1^·bar^−1^, which is 4–50 times less than the permeability of such commercial membranes as NF-90, NF-270, NF-2, NFPES-10 [55]. This shows the development paths of the studied polymeric alginate material. One solution to this problem is to create mixed matrix membranes. It is known from the literature that the introduction of, for example, titanium dioxide [30], carbon nanotubes [29] can significantly increase the water permeability to *P*_H2O_ ≥ 20 kg·m^−2^·h^−1^·bar^−1^ of alginate membranes.

Comparison of the nanofiltration properties of the obtained membranes with the characteristics of commercial membranes are presented in Table 4.

## 4. Conclusions

In this work, a biodegradable natural material (sodium alginate) was investigated to obtain composite membranes used in the nanofiltration separation of substances simulating antibiotics from organic and aqueous media. Organic protic and aprotic solvents—ethanol, toluene, hexane, and acetone—were used as precipitants to prepare nanofiltration composite membranes based on sodium alginate by the NIPS method for the first time. It was shown that NaAlg is formed due to strong sorption in toluene and hexane, and completely precipitated polymer is formed in ethanol and acetone. Protic ethanol and aprotic toluene were chosen to obtain OSN membranes. Such composite membranes showed low rejection of <10% for the model substance Remazol Brilliant Blue R with *MW* = 626 g/mol. To increase the separating properties of the composite membranes after deposition, they were crosslinked with metal cations with different valences. For this, traditional crosslinking agent—an aqueous solution of CaCl_2_—was used. In addition, aqueous solutions of the salts AlCl_3_ and AgNO_3_ were used for the first time. Composite membranes were also obtained with simultaneous precipitation and crosslinking with aqueous solutions of metal salts without using organic solvents. Composite membranes based on silver and aluminum alginate, formed by simultaneous precipitation and crosslinking in aqueous solutions of AlCl_3_ and AgNO_3_ salts, demonstrated the best properties in nanofiltration of ethanol+ Remazol Brilliant Blue R. The rejection rates of the model dyes were 93 and 90%, respectively. Crosslinking of composite membranes precipitated in organic precipitators with metal cations did not allow reaching a rejection of more than 40% from ethanol.

However, NaAlg composite membranes precipitated with organic solvents and then crosslinked with Ca^2+^, Al^3+^, and Ag^+^ metal cations showed the best performance in aqueous media filtration. All composite membranes obtained by precipitation in toluene had rejection Orange II (*MW* = 350 g/mol) and Remazol (*MW* = 626 g/mol) above 95 and 99%, respectively. Water permeability varied from 0.44 to 7.8 kg m^−2^h^−1^ bar^−1^ depending on the alginate cation. Ethanol precipitated AgAlg composite membranes had the best rejection of 99% for both model substances at a water permeability of 3.5 kg m^−2^ h^−1^ bar^−1^. Thus, it was shown that due to the variation of the precipitant and the crosslinking agent, the necessary properties of nanofiltration composite membranes could be selected. It makes cheap and biodegradable sodium alginate promising and versatile for isolating antibiotics from their extractants in pharmaceutical production and removing the same antibiotics from aqueous pharmaceutical effluents to prevent their accumulation in the environment the emergence of resistant genes and bacteria.

## Figures and Tables

**Table 1:** Solubility parameters according to Hansen [43] and according to Hildebrant for sodium alginate [23,44,45].

Compound	*δ*_t_, MPa^1/2^	*δ*_d_, MPa^1/2^	*δ*_p_, MPa^1/2^	*δ*_h_, MPa^1/2^	Ref.
NaAlg	37	-	-	-	[23,44,45]
Water	47.8	15.5	16.0	42.3	[43]
Ethanol	26.5	15.8	8.8	19.4	
Acetone	19.9	15.5	10.4	7.0	
Hexane	14.9	14.9	0.0	0.0	
Toluene	18.2	18.0	1.4	2.0	

**Table 2:** Permeability of composite membranes based on sodium alginate precipitated in organic solvents.

Precipitant	*K*_s (precipitant)_, g/g	Thinkeness of Swollen NaAlg Layer, µm	NaAlg Layer Appearance	Photo Precipitation NaAlg	*P,* kg∙m^−2^·h^−1^·bar^−1^			
					Toluene	Hexane	Ethanol	Acetone
Toluene	0.24	110	gel	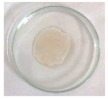	415	123	23	28
Hexane	0.11	80	gel	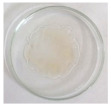	41	23	12	9
Ethanol	<0.05	55	precipitated polymer	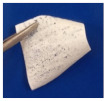	3	4	9	7
Acetone	<0.05	40	precipitated polymer	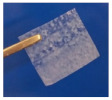	0.1	0.4	3.4	3.7

**Table 3:** Atomic concentrations of metals (%) in selective layers of composite membranes based on sodium alginate.

	Water				EtOH				Toluene			
	Na^+^	Al^3+^	Ca^2+^	Ag^+^	Na^+^	Al^3+^	Ca^2+^	Ag^+^	Na^+^	Al^3+^	Ca^2+^	Ag^+^
Na, %	10.4 ± 2.3	0	0.4± 0.1	0	11.2 ± 2.3	0	0.3± 0.1	0	10.9 ± 2.3	0	0.5± 0.1	0
Al, %	0	6.4 ± 2.2	0	0	0	7.7 ± 2.2	0	0	0	7.1± 2.2	0	0
Ca, %	0	0	6.2 ± 1.3	0	0	0	7.5 ± 1.3	0	0	0	5.9 ± 1.3	0
Ag, %	0	0	0	7.7 ± 0.2	0	0	0	7.9 ± 0.2	0	0	0	8.2 ± 0.2

**Table 4:** Comparison of transport and separation characteristics formed in the work of membranes and literature data.

Membrane Type	Filtered Media	*P*, kg·m^−2^·h^−1^·bar^−1^	Selectable Component	MW, g/mol	R, %	Ref.
CaAlg	MeOH	1.3–1.6	Vitamin B12	1355	90–98	[10]
MPF-44	EtOH + Methylene Blue	0.1	Methylene Blue	374	95	[53]
MPF-34		0.1			94	
M4		0.5			97	
Starmem		4.3			43	
Solsep		7.1			70	
Cellophane	EtOH	0.05	Remazol Brilliant Blue R	626	79	[56]
			Orange II	350	55	
AlAlg	EtOH	2.6	Remazol Brilliant Blue R	626	93	This work
AgAlg		1.5			90	
alginate crosslinked + PVA	water + PEG600	4	PEG600	600	80	[54]
AlAlg	water	7.8	Remazol Brilliant Blue R	626	99	This work
			Orange II	350		
AgAlg		6.8	Remazol Brilliant Blue R	626	99	
			Orange II	350	95	
Cellophane	water	0.11	Remazol Brilliant Blue R	626	100	[56]
			Orange II	350	97	

## Data Availability

Data sharing is not applicable.

## Supplementary Materials

The following supporting information can be downloaded at: https://www.mdpi.com/article/10.3390/membranes12020235/s1, Figure S1: The structural formula of sodium alginate. Figure S2: Schematic structural formulas (A) NaAlg; (B) CaAlg; (C) AlAlg. Figure S3: Model substances of antibiotics: A—Remazol Briliant Blue R (MW = 626 g/mol), B—Orange II (MW = 350 g/mol). Figure S4: The scheme of membranes preparation. Figure S5: Structural formulas of simulated antibiotics.

## List of Symbols and Acronyms

MW	molecular weight;
NaAlg	sodium alginate;
OSN	organic solvent nanofiltration;
API	active pharmaceutical ingredients;
WHO	World Health Organization;
CaCl_2_	calcium chloride;
AlCl_3_	aluminum chloride;
AgNO_3_	silver nitrate;
IR	infrared spectroscopy;
ATR	attenuated total reflection;
MWD	molecular weight distribution;
GPC	gel permeation chromatography;
NaCl	sodium chloride;
NIPS	non–solvent induced phase separation;
*P*	permeance, kg/m^2^ h bar;
m	weight of permeate, kg;
S	area of membrane, m^2^;
Δt	filtration time, h;
Δp	pressure drop, bar;
*R*	rejection, %;
C_p_	dye concentrations in the permeate;
C_0_	dye concentrations in the feed;
EDX	dispersive elemental spectroscopy;
XRD	X-ray diffraction;
K_s_	sorption, %;
m_0_	weight of the dry sample of membrane;
m_1_	weight of the swollen sample of membrane;
SEM	scanning electron microscopy;
DMF	dimethylformamide;
NMP	N–methyl–2–pyrrolidone;
EtOH	ethanol;
*η*	viscosity, mPa∙s;
ω_precipitant_	concentration of precipitant in alginate solution, %;
*δ*_t_	Hildebrand parameter;
*δ*_d_	dispersion component of Hildebrand parameter;
*δ*_p_	polar component of Hildebrand parameter;
*δ*_h_	hydrogen bond component of Hildebrand parameter;
AgAlg	silver alginate;
CaAlg	calcium alginate;
AlAlg	aluminum alginate.
