# Supplementary material for: Recovery of Model Pharmaceutical Compounds from Water and Organic Solutions with Alginate-Based Composite Membranes
Source: Membranes (Basel). 2022 Feb 18;12(2):235. doi: 10.3390/membranes12020235 (PMC8876430; doi:10.3390/membranes12020235)
Supplement: Supplementary file 1 [file membranes-12-00235-s001.zip › membranes-1543219-supplementary.pdf]

Supplementary

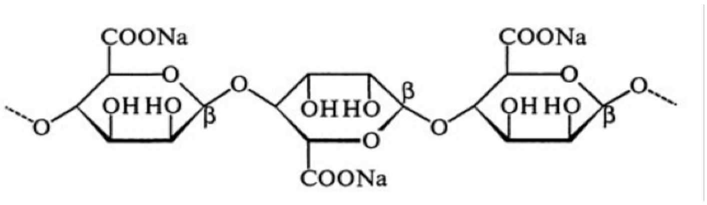

Figure S1. The structural formula of sodium alginate.

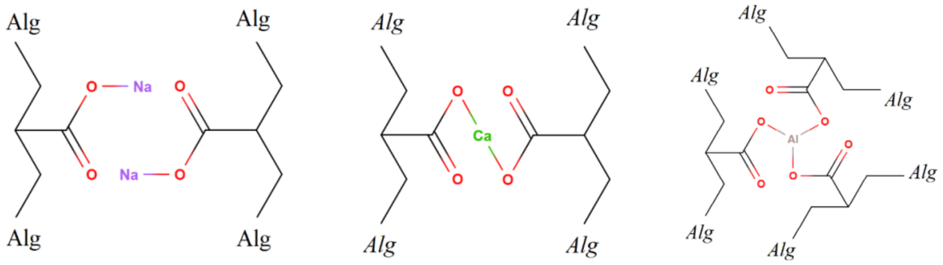

A B C

Figure S2. Schematic structural formulas A) NaAlg; B) CaAlg; C) AlAlg.

Supplementary 3

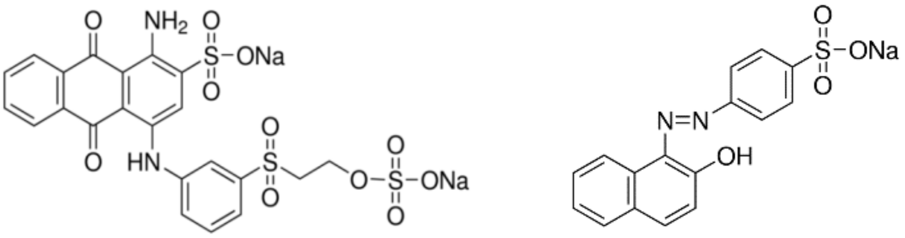

a B

Figure S3. Model substances of antibiotics: A - Remazol Briliant Blue R (MW = 626 g/mol), B - Orange II (MW = 350 g/mol).

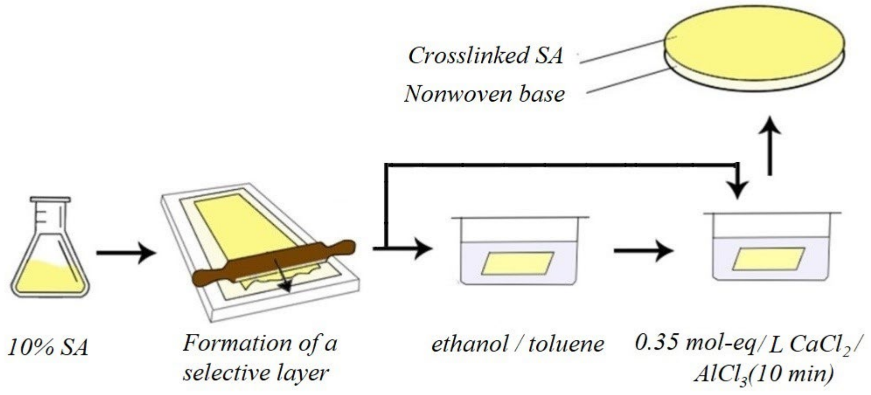

Figure S4. The scheme of membranes preparation.

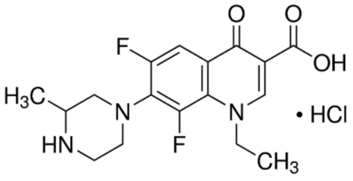

Lomefloxacin
(MW = 351 g/mol)

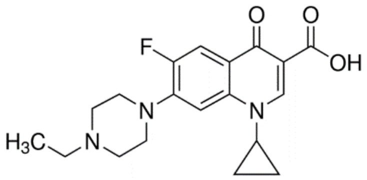

Enrofloxacin
(MW = 359 g/mol)

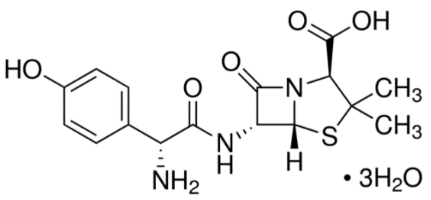

Amoxicilin
(MW = 365 g/mol)

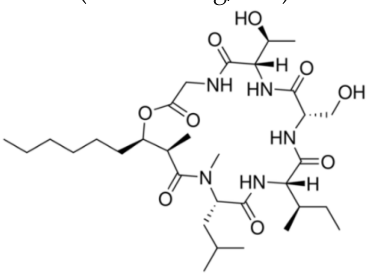

Globomycin
(MW = 655 g/mol)

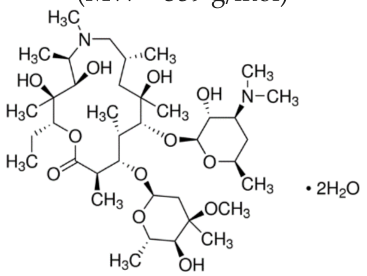

Azithromycin
(MW = 749 g/mol)

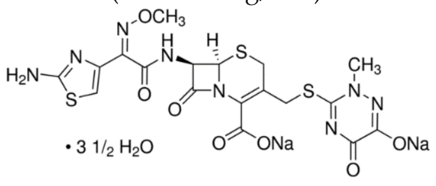

Ceftriaxone
(MW = 661 g/mol)

Figure S5. Structural formulas of simulated antibiotics.
